# Development and validation of mathematical nomogram for predicting the risk of poor sleep quality among medical students

**DOI:** 10.3389/fnins.2022.930617

**Published:** 2022-09-23

**Authors:** Jiahao Ding, Xin Guo, Mengqi Zhang, Mingxia Hao, Shuang Zhang, Rongshen Tian, Liting Long, Xiao Chen, Jihui Dong, Haiying Song, Jie Yuan

**Affiliations:** ^1^School of Psychology and Mental Health, North China University of Science and Technology, Tangshan, China; ^2^Department of Neurology, The First Hospital of Hebei Medical University, Shijiazhuang, China; ^3^Jitang College of North China University of Science and Technology, Tangshan, China

**Keywords:** nomogram, medical students, sleep quality, prediction model, PSQI

## Abstract

**Background:**

Despite the increasing prevalence of poor sleep quality among medical students, only few studies have identified the factors associated with it sing methods from epidemiological surveys. Predicting poor sleep quality is critical for ensuring medical Students’ good physical and mental health. The aim of this study was to develop a comprehensive visual predictive nomogram for predicting the risk of poor sleep quality in medical students.

**Methods:**

We investigated medical Students’ association with poor sleep quality at JiTang College of North China University of Science and Technology through a cross-sectional study. A total of 5,140 medical students were randomized into a training cohort (75%) and a validation cohort (25%). Univariate and multivariate logistic regression models were used to explore the factors associated with poor sleep quality. A nomogram was constructed to predict the individual risk of poor sleep quality among the medical students studied.

**Results:**

31.9% of medical students in the study reported poor sleep quality. We performed multivariate logistic analysis and obtained the final model, which confirmed the risk and protective factors of poor sleep quality (*p* < 0.05). Protective factors included the absence of physical discomfort (OR = 0.638, 95% CI: 0.546–0.745). Risk factors included current drinking (OR = 0.638, 95% CI: 0.546∼0.745), heavy study stress (OR = 2.753, 95% CI: 1.456∼5.631), very heavy study stress (OR = 3.182, 95% CI: 1.606∼6.760), depressive symptoms (OR = 4.305, 95% CI: 3.581∼5.180), and anxiety symptoms (OR = 1.808, 95% CI: 1.497∼2.183). The area under the ROC curve for the training set is 0.776 and the area under the ROC curve for the validation set is 0.770, which indicates that our model has good stability and prediction accuracy. Decision curve analysis and calibration curves demonstrate the clinical usefulness of the predictive nomograms.

**Conclusion:**

Our nomogram helps predict the risk of poor sleep quality among medical students. The nomogram used includes the five factors of drinking, study stress, recent physical discomfort, depressive symptoms, and anxiety symptoms. The model has good performance and can be used for further research on and the management of the sleep quality of medical students.

## Introduction

Poor sleep quality is an important contributing factor to adults’ decreased physical and mental health. Many harmful problems such as heart disease, psychological changes, high blood pressure, diabetes, weight gain, and more are linked to a lack of sleep (Bin Heyat et al., 2021). Previous epidemiological and clinical studies have shown that sleep has many functions that range from muscle damage recovery (Chennaoui et al., 2021) to brain metabolite clearance (Xie et al., 2013). Therefore, maintaining good sleep can promote better memory retention and (Rasch and Born, 2013) and improve cognitive function, and it can also help prevent age-related cognitive decline (Scullin and Bliwise, 2015). Research shows that sleep conditions reflect changes in brain development, and sleep can affect brain plasticity during critical periods (Wintler et al., 2020). Good sleep not only fully consolidates memory but also helps regulate emotions (Paller et al., 2021). However, currently, poor sleep quality plagues nearly a quarter of the world’s population (Fang et al., 2019). The incidence of poor sleep quality increased significantly during the COVID-19 pandemic, and a meta-analysis showed that the combined incidence of poor sleep quality among the general population reached 34% (Deng et al., 2021).

Poor sleep quality, whether short-term or chronic, is a common condition. It can negatively impact vulnerable patient populations (Dopheide, 2020). Previous research suggests that both young and old populations should get 7–9 h of sleep each night (Maheshwari and Shaukat, 2019), but, many people are sleep deprived (Kim et al., 2018). Approximately 33–50% of adults experience insomnia symptoms (Ancoli-Israel and Roth, 1999), and poor sleep quality is found to be 1.5 times more common in women than men (Suh et al., 2018). Insomnia is also a common health problem among contemporary college students (Lund et al., 2010). In a recent meta-analysis study conducted on Chinese college students, the percentage of students who sleep less than 6 and 7 h/d is 8.4 and 43.9%, respectively, revealing that short sleep time and unhealthy sleep patterns are very common among Chinese college students (Li et al., 2017). Other studies also show that lack of sleep has a negative impact on college Students’ academic performance and life (Suardiaz-Muro et al., 2020).

Like many college students, medical students also suffer from sleep deprivation (Christodoulou et al., 2021). A large contributing factor toward medical Students’ lack of sleep is poor sleep quality. Medical students face severe depression and anxiety (Rotenstein et al., 2016) and poor sleep quality (Azad et al., 2015) due to difficulties such as long learning cycles, heavy academic pressure (Ranasinghe et al., 2017), less physical activity (Luciano et al., 2021), and high clinical practice load (Dyrbye and Shanafelt, 2016). A meta-analysis by Aamir has confirmed that chronic physical inactivity affects sleep quality in medical students (Memon et al., 2021). Another meta-analysis also showed that study stress affects medical Students’ sleep quality (Memon et al., 2021). Therefore, problems of poor sleep quality and lack of sleep are common among medical students (Almojali et al., 2017). At the same time, the sleep quality of young people is related to gender, and the incidence of women’s poor sleep quality in the general population is higher than that in men (Fatima et al., 2016). However, there are also studies showing that poor sleep quality in medical students is not related to gender (Chen et al., 2020). A large number of studies have shown that depression has a relationship with sleep quantity and quality, and 90% of depressed patients have complained about sleep quality problems (Tsuno et al., 2005). One study showed that the average prevalence of depression among Chinese medical students was 32.74%, while the average prevalence of anxiety was 27.22% (Mao et al., 2019). Additionally, medical students with poor sleep are more than three times more likely to report depression than other college students (Jin et al., 2021). Meanwhile, a recent meta-analysis of sleep quality among medical students also showed that anxiety and depression were highly associated with poor sleep quality (Rao et al., 2020). Interventions for depression may therefore improve sleep quality (Wichniak et al., 2017). Although poor sleep quality among medical students has been widely studied and reported (Almojali et al., 2017; Chen et al., 2020; Christodoulou et al., 2021; Mazar et al., 2021), we still lack an effective tool to screen medical students for poor sleep quality.

Nomograms have been widely used as a reliable clinical tool to create a simple intuitive graph for quantifying the risk of a clinical event of interest, based on multiple logistic regression or Cox Proportional Hazards regression model (Li et al., 2020). With the ability to generate an individual numerical probability of a clinical event by integrating diverse prognostic and determinant variables, it fulfills our desire for biologically and clinically integrated models and our drive toward personalized medicine (Balachandran et al., 2015).

Therefore, we conducted this study to elucidate and analyze the influencing factors of sleep quality, allow medical professionals in primary care settings to easily and visually identify symptoms of poor sleep, and construct a nomogram to predict the risk of poor sleep quality in medical students.

## Materials and methods

### Participants

This study was conducted between December 2020 and January 2021 in Tangshan City, Hebei Province, and students were selected from JiTang College of North China University of Science and Technology. This study adopted a random sampling method. The data collection period was from December 10, 2020, to January 10, 2021. Data collection via web platform. Participants must be enrolled at a university and have agreed to participate in this study. A cross-sectional survey of medical students was conducted of 5,993 college students across five grades. Data was entered by two different researchers using a double-blind input database for accuracy. We excluded 853 participants for ineligibility, including participants whose data did not meet the specifications, was incomplete, and those who were non-medical professionals.

A total of 5,140 valid questionnaires were finally screened, with a recovery rate of 85.8%. These participants were randomized into a training cohort (75%) and a validation cohort (25%). This survey was organized and coordinated by JiTang College of North China University of Science and Technology. All participants signed the online informed consent form and completed the online questionnaire. The study was then approved by the Biomedical Ethics Committee of JiTang College of North China University of Science and Technology (NCUSTJC-2021-0001). Considering that COVID-19 is still in its pandemic phase, the data was collected through online platforms rather than face-to-face interviews. All questionnaires were completed on the Questionnaire Star platform.

### Demographic basics

Questions asked to the participants included gender, age, ethnicity, grade, sexual orientation, relationship status, major, professional satisfaction, personality self-assessment, father’s education level, mother’s education level, drinking habits, smoking habits, weight control, and study pressure. Gender was defined as either male or female. The age groups were under 18, 18–22, 22–25, and over 25. Ethnic groups were divided into Han nationalities or ethnic minorities. Grades were divided into freshman, sophomore, junior, senior, and fifth. Sexual orientation was divided into heterosexual, homosexual, and bisexual. Relationship status was divided into married, in a relationship, single, and other. The majors were divided into clinical medicine, stomatology, traditional Chinese medicine, nursing, medical imaging, and pharmacy. Personality self-evaluation was divided into introverted and extroverted. Smoking was divided into never smoking, current smoking, and currently quitting smoking. Drinking was divided into never drinking and drinking. Study pressure was divided into very light, light, heavy, and very heavy.

It also included questions on mother’s education level, father’s education level, participants’ relationships with their parents, and any recent physical discomfort. The education level of mothers was divided into junior high school education and below, high school education, and college education and above. The education level of fathers was divided into junior high school education and below, high school education, college education, and above. Relationships with parents were classified as very good, good poor, and very poor. Recent physical discomfort was divided into yes and no.

### International physical activity questionnaire

In 1998, a group of experts developed the International Physical Activity Questionnaire (IPAQ), consisting of four short and four long versions, to be used for global standard physical activity monitoring (Craig et al., 2003). The short version records activity at four intensity levels: (1) high-intensity activities such as aerobics, (2) moderate-intensity activities such as recreational cycling, (3) walking, and (4) sitting (Lee et al., 2011). The IPAQ-SF defines the number of days and times during the past 7 days as high intensity, moderate intensity, or walking for at least 10 min. It also records the time spent sitting during the past 7 days on weekdays (Craig et al., 2003). The IPAQ-SF data was converted to metabolic equivalent minutes per week (MET-min/week) using the formula published by Hagströmer et al. (2006). Respondents who met certain criteria were considered active. This included at least 20 min of vigorous activity per day for ≥ 3 days, at least 30 min of moderate-intensity activity per day for ≥ 5 days, or any combination of ≥ 5 days of walking, moderate-intensity, and vigorous-intensity activity reaching at least 600 MET-min/week. Further, participants were also considered active if they participated in vigorous-intensity activity for at least 3 days and cumulative at least 1,500 MET-min/week or ≥ 7 days of walking in any combination to achieve at least 3,000 MET-min/week of moderate- or vigorous-intensity activity. Those who did not meet the above criteria were classified as inactive (Hagstromer et al., 2010). This study divided physical activity levels into three groups, high physical activity level, moderate physical activity level, and low physical activity level (Puciato et al., 2017).

### Measure of depression severity

The Patient Health Questionnaire-9 (PHQ-9) is a reliable and valid self-assessment questionnaire that measures the frequency of depressive symptoms in clinical practice and research over the past 2 weeks (Costantini et al., 2021). The PHQ-9 score is divided into 5 groups: 0–4, 5–9, 10–14, 15–19, and 20–27, corresponding to no, mild, moderate, moderate to severe, and severe depression, respectively (Kroenke et al., 2001). In this study, the results were largely divided into no a depressive symptom group (0–4) and a depressive symptom group (5–27).

### Generalized anxiety disorder screener

The Generalized Anxiety Disorder Scale-7 (GAD-7) is a 7-item self-rating scale developed by Spitzer as a screening tool and indicator of severity for generalized anxiety disorder (Spitzer et al., 2006; Rutter and Brown, 2017). The 7-item GAD-7 is widely considered to have good reliability, standard validity, construct validity, factorial validity, and procedural validity (Rutter and Brown, 2017). A recent study showed that the GAD-7 questionnaire can be administered rapidly, improving clinical screening time efficiency and maintaining good sensitivity and specificity to diagnose the most common anxiety disorders in primary care (Sapra et al., 2020). Each item ranges from 0 to 3 (“none at all,” “a few days,” “more than half,” “almost every day”). The total score ranges from 0 to 21, with higher total scores indicating greater GAD severity. According to the scoring standard, GAD-7 scores are divided into 4 groups: 0–5, 6–9, 10–14, and 15–21, corresponding to no, mild, moderate, and severe anxiety (Schalet et al., 2014). This study mainly divided the results into a no anxiety symptom group (0–4) and anxiety symptom group (5–27).

### Family affluence scale

The Family Affluence Scale (FAS) is an objective measure of national wealth to assess the wealth of young people’s households. This is required due to the difficulties young people often have in reporting household income (Boyce et al., 2006). Each Student’s composite FAS score is based on their response to the four items. According to the scoring scale, a low FAS score (0, 1, 2) means low wealth, an FAS medium score (3, 4, 5) means moderate wealth, and a high FAS score (6, 7, 8, 9) means rich (Currie et al., 1997).

### Pittsburgh sleep quality index

The Pittsburgh Sleep Quality Index (PSQI) is one of the most widely used sleep metrics in the world (Buysse et al., 1989). It is a sleep assessment method developed to help clinicians and researchers. The questionnaire is divided into seven components: Sleep quality, sleep onset latency, sleep duration, sleep efficiency, sleep disturbance, use of sleep medication, and daytime dysfunction (Farah et al., 2019). Each item is scored from 0 to 3. The total score for the seven components is called the PSQI score and ranges from 0 to 21. An overall PSQI score of more than 5 indicates poor sleep relative to clinical and laboratory measures, with higher scores indicating poorer sleep quality (Buysse et al., 1989). This study broadly divided the results into good sleep quality (0–5) and poor sleep quality (6–21).

### Statistical analysis

Statistical differences in age distributions in the training and validation sets were assessed using the Wilcoxon test, and other changes were analyzed using the chi-square test with *P* < 0.05 considered statistically significant. Multivariate logistic regression analysis was performed on variables that had *p*-values less than 0.05 in the univariate logistic analysis. Statistical analysis was performed using SPSS software version 22.0 to identify risk factors. Based on the results of the multivariate analysis, a nomogram was developed using the r package “rms” in R software version 3.5.2.^1^ The performance of the nomogram was measured by the concordance index (C-index), which is equivalent to the area under the curve value (AUC). It is evaluated by comparing the predicted probability of the nomogram with its observed probability. The calibration was assessed by comparing the nomogram predicted probability with the actual probability, which was visualized by calibration curves plot using 1,000 bootstrap resamples procedures, tested by Hosmer-Lemeshow test and the variance inflation factor (VIF). The C-index reflects the probability, as predicted by the nomogram, of a randomly selected student with a higher risk of poor sleep quality being more likely to have sleep disturbance than another student with a lower predicted risk. A higher C-index indicates improved ability to discriminate between different college students with poor sleep quality. Calibration curves were used to compare predicted probabilities and observed probabilities in the study. If the model is properly calibrated, the points on the calibration map should be close to the 45° diagonal.

## Results

### Participant inclusion

**FIGURE 1:**
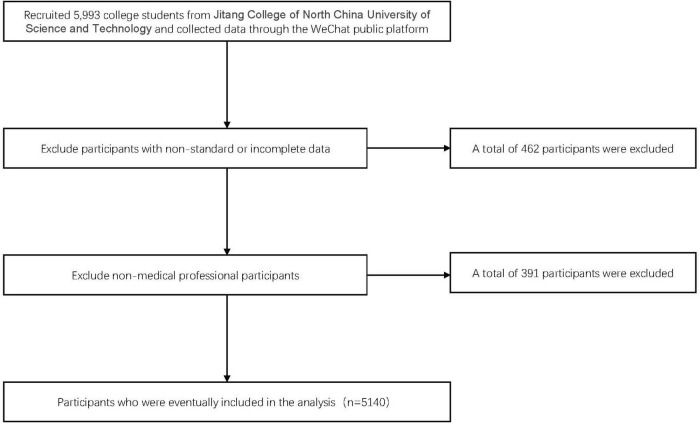
Flow chart of sample selection according to inclusion and exclusion criteria.

A total of 5,993 college students from JiTang College of North China University of Technology were recruited for this study. We excluded 462 participants with non-standard or incomplete data and 391 participants who were not medical professionals, resulting in a total of 5,140 participants (Figure 1).

### Participant characteristics

In this study, 31.9% medical students in the total sample (*n* = 3,855) reported poor sleep quality. The total number of samples (*n* = 5,140) was divided into a training set (*n* = 3,855) and a validation set (*n* = 1,285) in the ratio of 3:1. The baseline levels of sample characteristics of the training set and validation set are shown in Table 1. In the training set of this study, 67.7% (2,608/3,855) of participants had no poor sleep quality and 32.3% (1,247/3,855) had poor sleep quality. In the validation set, 69.6% (894/1,285) of participants did not report poor sleep quality and 30.4% (391/1,285) did. No significant differences were observed in the baseline characteristics of subjects between the training and validation groups (*p*-value range from 0.11 to 1.000) (Table 1).

**TABLE 1:** Demographic and clinical characteristics of medical students.

Predictors	Total	Training set	Validation set	*P*
				
	(*N* = 5,140)	(*N* = 3,855)	(*N* = 1,285)	
**Sleep quality**				
Good	3,502 (68.1%)	2,608	894	0.210
Poor	1,638 (31.9%)	1,247	391	
**Gender**				
Men	1,826 (35.5%)	1,378	448	0.590
Women	3,314 (64.5%)	2,477	837	
Age (years)				0.720
<18	44 (0.9%)	33	11	
18–21	3,587 (69.8%)	2,675	912	
22–25	1,506 (29.3%)	1,142	364	
>25	11 (0.2%)	9	2	
**Major**				
Clinical medicine	2,483 (48.3%)	1,869	614	0.970
Stomatology	496 (9.6%)	372	124	
Traditional Chinese medicine	651 (12.7%)	485	166	
Nursing	1,007 (19.6%)	754	253	
Medical imaging	220 (4.3%)	160	60	
Pharmacy	283 (5.5%)	215	68	
**Character self-assessment**				
Introverted	3,243 (63.1%)	2,444	799	0.450
Extrovert	1,897 (36.9%)	1,411	486	
Smoking				0.110
Don’t smoking	4,798 (93.4%)	3,590	1,208	
Currently smoking	237 (4.6%)	177	60	
Quitting smoking	105 (2.0%)	88	17	
**Drinking**				1.000
Don’t drinking	3,526 (68.6%)	2,644	882	
Currently drinking	1,614 (31.4%)	1,211	403	
**Study stress**				
Very light	124 (2.4%)	92	32	0.190
Light	3,317 (64.5%)	2,457	860	
Heavy	1,368 (26.6%)	1,053	315	
Very heavy	331 (6.4%)	253	78	
**Feeling unwell**				
Yes	2,113 (41.1%)	1,586	527	0.960
No	3,027 (58.9%)	2,269	758	

The comparison of characteristics of the poor sleep quality and good sleep quality medical students were presented. The occurrence of poor sleep quality was influenced by factors such as professional satisfaction, personality self-assessment, drinking, study stress, feeling unwell, depression, and anxiety. In the training set, as shown in Table 2, participants who were very dissatisfied with their occupation had a higher risk of poor sleep quality (*P* = 0.000). Compared with non-drinkers, chronic alcohol drinkers had a higher risk of poor sleep quality (*P* < 0.000). Heavy study pressure significantly increased the risk of poor sleep quality among medical students (*P* = 0.000). Participants with no recent physical distress had an increased risk of poor sleep quality (*P* = 0.000). Medical students with depressive symptoms (*P* = 0.000) and anxiety symptoms (*P* = 0.000) had a significantly higher incidence of poor sleep quality. The same depression demographics were also shown in the validation cohort (see Supplementary Appendix and Table 2).

**TABLE 2:** The comparison of characteristics of the sleep quality medical students were presented in the training set.

	Total (*N* = 3,855)	Poor sleep quality		*P*
			
		No (*N* = 2,608)	Yes (*N* = 1,247)	
Professional satisfaction				*p* < 0.001
Very satisfied	1,469	1,080 (73.5%)	389 (26.5%)	
Satisfy	1,502	1,004 (66.8%)	498 (33.2%)	
Ordinary	794	475 (59.8%)	319 (40.2%)	
Dissatisfied	56	31 (55.4%)	25 (44.6%)	
Very dissatisfied	34	18 (52.9%)	16 (47.1%)	
Drinking				*p* < 0.001
Don‘t drinking	2,644	1,845 (69.8%)	799 (30.2%)	
Drinking	1,211	763 (63.0%)	448 (37.0%)	
Study stress				*p* < 0.001
Very light	92	80 (87.0%)	12 (13.0%)	
Light	2,457	1,802 (73.3%)	655 (26.7%)	
Heavy	1,053	601 (57.1%)	452 (42.9%)	
Very heavy	253	125 (49.4%)	128 (50.6%)	
Feeling unwell				*p* < 0.001
Yes	1,586	892 (56.2%)	694 (43.8%)	
No	2,269	1,716 (75.6%)	553 (24.4%)	
Depression				*p* < 0.001
No	2,218	1,883 (84.9%)	335 (15.1%)	
Yes	1,637	725 (44.3%)	912 (55.7%)	
Anxiety				*p* < 0.001
No	2,757	2,159 (78.3%)	598 (21.7%)	
Yes	1,098	449 (40.9%)	649 (59.1%)	

### Nomogram variable screening

According to the results of the univariate logistic regression analysis, sleep disorders in medical students were significantly correlated with their majors, alcohol drinking habits, recent study pressure, recent physical discomfort, physical activity intensity, depression symptoms, and anxiety symptoms (*p* < 0.05). The participants’ recent physical good and high- and moderate-intensity physical activity were protective factors for sleep disorders in medical students. Independent risk factors for sleep disorders among medical students included whether the participants were in their recent drinking habits (OR = 1.356, 95% CI: 1.175–1.564), whether they underwent heavy learning stress (OR = 5.014, 95% CI: 2.806–9.783) or very heavy learning stress (OR = 6.827, 95% CI: 3.670–13.741), and whether they exhibited depressive symptoms (OR = 7.071, 95% CI: 6.081–8.238) and had anxiety (OR = 5.219, 95% CI: 4.491–6.070). Because there were more than three risk factors in univariate logistic regression analysis, the interaction of influencing factors had to be carefully avoided. To achieve this, the influencing factors screened by univariate analysis were included in multivariate logistic regression analysis, and the relationship between sleep disturbance and risk factors such as depression and anxiety was finally determined (Table 3).

**TABLE 3:** Univariate and multivariate logistic analysis of risk factors for poor sleep quality among medical students.

Predictors	Univariate analysis			Multivariate analysis		
		
	OR	95% Cl	*p*	OR	95% Cl	*p*
**Major**						
Clinical medicine	Ref	Ref	Ref	Ref	Ref	Ref
Stomatology	0.983	0.462∼0.559	0.889	1.064	0.810∼1.392	0.655
Traditional Chinese medicine	0.996	0.775∼1.243	0.967	0.934	0.732∼1.189	0.582
Nursing	0.739	0.613∼0.890	0.002	0.808	0.648∼1.006	0.058
Medical imaging	0.868	0.607∼1.224	0.428	0.98	0.651∼1.460	0,923
Pharmacy	1.054	0.780∼1.413	0.730	1.408	0.996∼1.979	0.051
**Drinking**						
Don’t drinking	Ref	Ref	Ref	Ref	Ref	Ref
Currently drinking	1.356	1.175∼1.564	*p* < 0.001	1.306	1.107∼1.540	0.002
**Study stress**						
Very light	Ref	Ref	ref	Ref	Ref	Ref
Light	2.423	1.365∼4.706	*p* < 0.001	1.924	1.010∼3.970	0.059
Heavy	5.014	2.806∼9.783	*p* < 0.001	2.71	1.413∼5.620	0.004
Very heavy	6.827	3.670∼13.741	*p* < 0.001	3.047	1.519∼6.554	0.003
**Feeling unwell recently**						
Yes	Ref	Ref	Ref	Ref	Ref	Ref
No	0.414	0.361∼0.475	*p* < 0.001	0.653	0.558∼0.764	*p*<0.001
IPAQ						
LPAL	Ref	Ref	Ref	Ref	Ref	Ref
MPAL	0.851	0.736∼0.985	0.030	0.884	0.748∼1.044	0.146
HPAL	0.803	0.651∼0.988	0.040	0.86	0.675∼1.092	0.217
**Depression**						
No	Ref	Ref	Ref	Ref	Ref	Ref
Yes	7.071	6.081∼8.238	*p* < 0.001	4.239	3.514∼5.121	*p*<0.001
**Anxiety**						
No	Ref	Ref	Ref	Ref	Ref	Ref
Yes	5.219	4.491∼6.070	*p* < 0.001	1.799	1.484∼2.179	*p*<0.001

### Nomogram construction and internal validation

**FIGURE 2:**
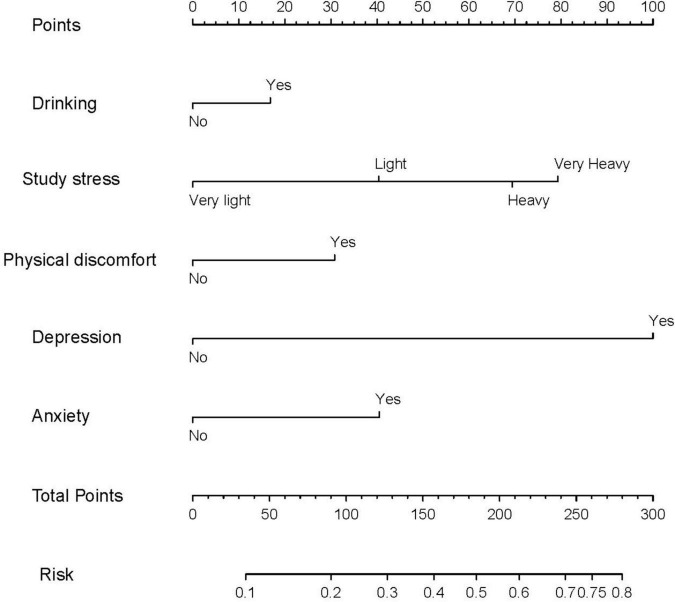
The prediction nomograms of risk factors for poor sleep quality in medical students.

**FIGURE 3:**
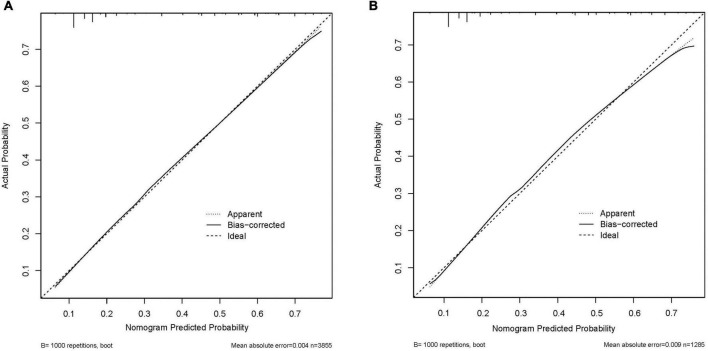
The logistic calibration curve of the prediction nomograms of risk factors for poor sleep quality in medical students. **(A)** Calibration curve of the poor sleep quality nomogram prediction in the training set. **(B)** Calibration curve of the poor sleep quality nomogram prediction in the validation set.

**FIGURE 4:**
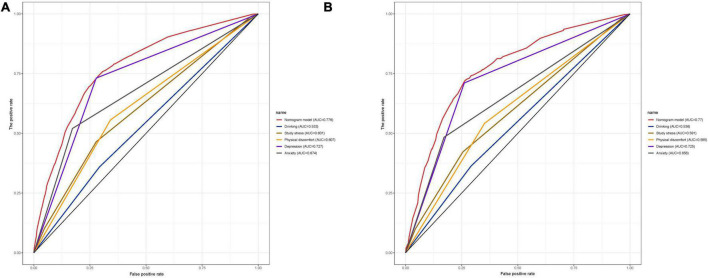
The ROC curve of the developed nomogram for predicting poor sleep quality in medical students. AUC, the area under the curve. **(A)** ROC curve of the training set nomogram. **(B)** ROC curve of the validation set nomogram.

**FIGURE 5:**
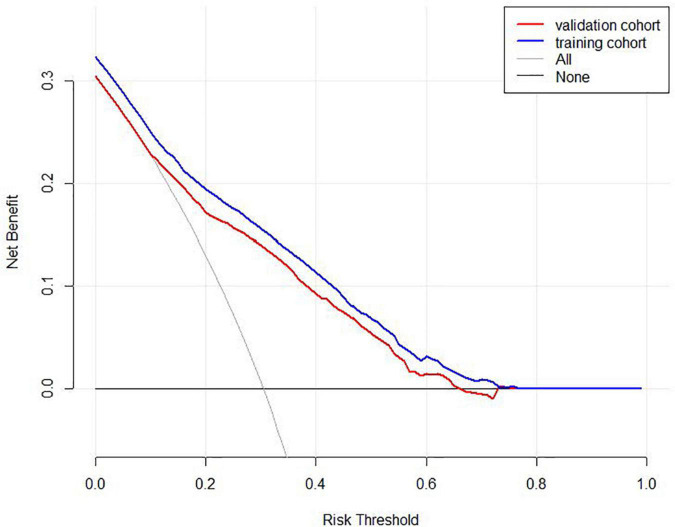
Decision curve analysis for the predictive model. The net benefit was produced against the high-risk threshold. The red solid line represents the validation cohort. The blue solid line represents the training cohort. The decision curve shows that the incidence of poor sleep quality among medical students is 31.9%, and at the threshold of 31.9%, the decision curve is above the None line and the All line, so the model has clinical utility.

We included the univariate logistic regression analysis results in the multivariate logistic regression and calculated the AIC value. When all factors were included in the multivariate regression, the AIC was 4011.65. After that, we adjusted the model to remove the two factors of majors and physical activity intensity, and the final model AIC was 4009.81. Finally, we built the final model (Table 4). An analysis of the final model confirmed the protective and risk factors for poor sleep quality in medical students (*p* < 0.05). Protective factors included the absence of physical discomfort (OR = 0.638, 95% CI: 0.546–0.745). Risk factors included heavy drinking habits (OR = 0.638, 95% CI: 0.546∼0.745), heavy study stress (OR = 2.753, 95% CI: 1.456∼5.631), very heavy study stress (OR = 3.182, 95% CI: 1.606∼6.760), depressive symptoms (OR = 4.305, 95% CI: 3.581∼5.180), and anxiety symptoms (OR = 1.808, 95% CI: 1.497∼2.183). Based on the final model, we constructed a nomogram based on the risk factors for poor sleep quality among recruited medical students (Figure 2). The following five indicators were selected: Drinking, study pressure, recent physical discomfort, depressive symptoms, and anxiety symptoms. The prediction model defines the first factor as no if not drinking and yes otherwise. Unless physical discomfort is defined as no, it is defined as yes. Subjects without depressive symptoms were defined as no or yes. Absence of anxiety symptoms was defined as 0 or 1. For example, in the nomogram, if the medical students do not drink alcohol, it is 0 points. If they are under heavy study pressure, it is 70 points. If they have had recent back discomfort, it is 30 points. Depressive symptoms would be 100 points and no anxiety symptoms are 0 points. In such an example, the total score would be 200 points. The nomograms (Figure 3) for both training and validation set calibration curves show that the points on the curves are all close to the 45° diagonal, with good agreement and a high degree of calibration. Moreover, the Hosmer–Lemeshow test indicated that the nomogram model had a satisfactory fit (*P* = 1.000). All predictors had no multicollinearity because the VIF in all them was < 1.429. This suggests that the model is more accurate. Subsequently, the ROC curves of the prediction model in the training and validation datasets were analyzed to evaluate the diagnostic effect of the model (Figure 4). The area under the ROC curve (AUC) for the training and validation sets were 0.776 and 0.770, respectively, which indicate that the model could accurately predict poor sleep quality in medical students. Decision curve analysis shows that the predictive model has a wide range of applications (Figure 5).

**TABLE 4:** The final model for predicting poor sleep quality among medical students.

Predictors	OR	95% Cl	*P*
**Drinking**			
Don’t drinking	Ref	Ref	Ref
Drinking	1.278	1.087∼1.503	0.003
**Study stress**			
Very light	Ref	Ref	Ref
Light	1.803	0.962∼3.667	0.082
Heavy	2.753	1.456∼5.631	0.003
Very heavy	3.182	1.606∼6.760	0.002
**Feeling unwell recently**			
Yes	Ref	Ref	Ref
No	0.638	0.546∼0.745	*p* < 0.001
**Depression**			
No	Ref	Ref	Ref
Yes	4.305	3.581∼5.180	*p* < 0.001
**Anxiety**			
No	Ref	Ref	Ref
Yes	1.808	1.497∼2.183	*p* < 0.001

Overall, the results of this study suggest that a nomogram constructed with the five factors listed above has accurate predictive value for the risk of poor sleep quality among medical students.

### Discussion

To our knowledge, this study is the first attempt to construct a nomogram that predicts the risk of poor sleep quality in medical students. We used five variables for this prediction. They are: Drinking, study stress, recent physical discomfort, depression, and anxiety. This can help medical staff better identify high-risk groups among medical students and provide them with any early help they might need. The nomogram also displayed excellent discrimination with AUC of 0.776 and good calibration. High AUC of 0.772 could still be reached in the internal validation. DCA and clinical impact curve showed that the majority of the threshold probabilities in this model had good net benefits.

This study used the PSQI to identify factors associated with poor sleep quality in medical students, and demonstrated that the incidence of poor sleep quality was 31.9%. This figure is consistent with many previous studies (Johnson et al., 2017; Eleftheriou et al., 2021; Gupta et al., 2021; Olarte-Durand et al., 2021).

Similar to previous studies (Chen et al., 2020), this study showed no statistically significant difference between poor sleep quality and gender. However, a previous study showed that women are almost twice as likely than men to have poor sleep quality (Madrid-Valero et al., 2017).

Previous studies have found that physical activity is generally thought to contribute to sleep, although this link may be influenced by multiple moderators such as gender, age, fitness level, sleep quality, and the intensity, duration, and environment of the exercise (Chennaoui et al., 2015). A meta-analysis of college Students’ sleep and physical activity found that most studies did not find a link between sleep and physical activity in college students (Memon et al., 2021), which is in accordance with the results of this study. Similarly, a study comparing the sleep quality of medical students and sports students also showed that there was no significant difference in the two (de Souza et al., 2021).

In this study, medical students in higher grades who exhibited drinking, high study stress, recent physical discomfort, poor relationships with their parents, and depressive and anxiety symptoms had a higher prevalence of poor sleep quality. Alcohol use is a risk factor for poor sleep quality, and while we cannot provide a causal explanation for the directional relationship between the two, our study and previous studies corroborate that poor sleep quality and greater alcohol-related outcomes are positively correlated (Kenney et al., 2012). In this study, sleep disturbance in college students was significantly related to study stress, which has been widely reported (Lund et al., 2010; Almojali et al., 2017; Safhi et al., 2020). Similarly, they also found a general decrease in sleep quality among students during exams (Ahrberg et al., 2012), Interestingly, alcohol consumption was found to decrease and caffeine consumption was found to increase during exams, which is a cause for concern (Ahrberg et al., 2012).

As expected, there was a strong association between sleep quality and depressive and anxiety symptoms. However, it is worth noting that the prevalence of depressive symptoms among medical students in this study was 41.8%, which is much higher than a meta-analysis on the prevalence of depression among college students (Ibrahim et al., 2013). This may be related to the fact that medical school is a stressful environment for students and Chinese clinical medical education consists of several different degrees and durations, including 3–8 year-long programs that award graduates a bachelor’s, master’s, or doctoral degree (Chen et al., 2020). This leaves medical students who have completed a 5-year undergraduate degree with the choice of either entering the workforce immediately or continuing on to a higher degree (Chen et al., 2020). The long-term education system of Chinese medicine makes Chinese medical students face both academic and economic pressure. Financial stress is thus also a possible cause of depression in medical students (Pham et al., 2019). Therefore, a large proportion of medical students, greater than the general population, report signs of mental illness (Puthran et al., 2016).

This study has some limitations. First, since this study is based on a single institution, the specific context of that institution may have an impact on the generalizability of the results. Future research should increase collaboration between research institutions so that the methodology can be applied to a wider range of contexts. Second, data collected at a single point in time may influence causal relationships between variables. Furthermore, although the questionnaires that did not immediately meet the requirements were excluded, recall bias may still exist in the answers studied.

### Conclusion

Our nomogram helps predict the risk of poor sleep quality among medical students. The nomogram used includes the five factors of drinking, study stress, recent physical discomfort, depressive symptoms, and anxiety symptoms. The model has good performance and can be used for further research on and the management of the sleep quality of medical students.

## Data availability statement

The raw data supporting the conclusions of this article will be made available by the authors, without undue reservation.

## Ethics statement

The studies involving human participants were reviewed and approved by the Jitang College of North China University of Science and Technology Experimental Ethics (NCUSTJC-2021-0001). Written informed consent from the participants’ legal guardian/next of kin was not required to participate in this study in accordance with the national legislation and the institutional requirements.

## Author contributions

JDi completed the design of the experiment, the writing of the manuscript, and the production of the chart. XG, MH, and MZ participated in the design of this review. SZ, RT, LL, XC, JDo, and HS completed data collection and screening. JY gave constructive guidance and made critical revisions. All authors contributed to the article and approved the submitted version.

## Footnotes

1
http://www.R-project.org/

## Abbreviations

IPAQ: International Physical Activity Questionnaire
PHQ-9: The Patient Health Questionnaire-9
GAD-7: The Generalized Anxiety Disorder Scale-7
FAS: The Family Affluence Scale
PSQI: The Pittsburgh Sleep Quality Index.
