# Supplementary material for: Development and validation of mathematical nomogram for predicting the risk of poor sleep quality among medical students
Source: Front Neurosci. 2022 Sep 23;16:930617. doi: 10.3389/fnins.2022.930617 (PMC9537862; doi:10.3389/fnins.2022.930617)
Supplement: Supplementary file 1 [file Data_Sheet_1.PDF]

```
##Comparison between groups
library(car)
library(survival)
library(rms)

{...}
#View the data (be sure to run this step)
if(TRUE){...}

##1.1 Set the number of cases of Group_A (number of rows first) and Group_B (number of rows last)
Group_A_number<-3855
Group_B_number<-1285
##1.2 Perform a test between Group_A and Group_B
{...}
##1.3 Test of interaction between variables within Group_A
{...}
```

##Ordinary Chi-Square Test
mytable <- xtabs(~a + b)
library(gmodels)
CrossTable(a, b)
chisq.test(mytable)

##logistics
library(car)
library(rms)
library(pROC)
library(DecisionCurve)

#Set the data set, just run the following lines
w<-data.frame(training_dataset)
str(w)
typeof(w)
ddist <- datadist(w)
options(datadist='ddist')

##Univariate Logistic Regression Analysis
w <-lrm(y~ x1, data=training_dataset, x=TRUE, y=TRUE,maxit=1000)

##View the single-factor Logistic analysis results, and the corresponding Coef and p values can be seen at the bottom.
print(w)

##After the completion of the single factor above, according to the results of the single factor analysis,
##combined with the research background, select the variables to be included in the multivariate regression, and modify the function here (connect the independent variables with

```
a + sign)
w <-lrm(y~ x1 + x2 + x3 + x4 + x5 + x6 + x7 + x8, data=training_dataset, x=TRUE,
y=TRUE,maxit=1000)
print(f_lrm)

#Use the backward variable selection method to filter variables.
fastbw(f, rule=c("aic"))

tlm<- glm(y~ x1 + x2 + x3 + x4 + x5 + x6 + x7 + x8 ,    family= binomial(), data=Example11_5)
tstep<-step(tlm)
summary(tstep)

#The following is a regression analysis based on the filtered variables
f <- lrm(y~ x1 + x2 + x3 + x4 + x5 + x6 + x7 + x8, data=w, x=TRUE, y=TRUE,maxit=5000)

#Check for collinearity among variables included in regression analysis
vif(f)

#Hosmer–Lemeshow
hoslem.test(training_dataset$y,predict(f,training_dataset),g=10)

#nomogram
nomogram <- nomogram(f,fun=function(x)1/(1+exp(-x)),
                     fun.at = c(0.01,0.1,0.3,0.5,0.8,0.9,0.99),
                     funlabel = "Prob of cluster 1",
                     lp=F,
                     conf.int = F,
                     abbrev = F
)
plot(nomogram)

pred_f_training<-predict(f_lrm,training_dataset)



#ROC
colnames(training_dataset)
#Set the parameters of each curve in the ROC plot
function_list<-c(
  "Death ~ Age+Gender+WHO_class_II_vs_I+WHO_class_III_vs_I",
  "Death ~ Age",
  "Death ~ Gender",
  "Death ~ WHO_class_II_vs_I+WHO_class_III_vs_I"
)
```

```
#Set the variable name of the ending event
outcome_name<-"Death"
#Set the name of each ROC
ROC_names_list<-c("Nomogram model","Age","Gender","WHO Class")
#Set the color of each ROC
ROC_color_list<-c("#CC3333", "#003399", "#996600", "#FF9900")
#The content in the brackets below runs directly
{
  ddist <- datadist(training_dataset)
  options(datadist='ddist')
  #Build a multifactor model
  lrm_list<-list()
  for(index in 1:length(function_list)){
    lrm_list[[index]]<-lrm(as.formula(function_list[index]), data=training_dataset, x=TRUE,
y=TRUE,maxit=5000)
  }
  #Calculate predicted values and ROC curve values
  pred_f_training<-list()
  modelroc<-list()
  for(index in 1:length(function_list)){
    pred_f_training[[index]]<-predict(lrm_list[[index]],training_dataset)
    modelroc[[index]] <- roc(training_dataset[,outcome_name],pred_f_training[[index]])
  }
  #Calculate AUC and add to name
  ROC_names_list_training<-ROC_names_list
  for(index in 1:length(function_list)){
    ROC_names_list_training[index]<-paste0(ROC_names_list_training[index],"
(AUC=",round(modelroc[[index]]$auc[[1]],3),")")
  }
  names(modelroc)<-ROC_names_list_training
  #Plot multiple ROC functions
  roc_list<-ggroc(modelroc, legacy.axes=TRUE,size = 1)+
    scale_colour_manual(values = ROC_color_list)+
    annotate(geom = "segment",x=0,y=0,xend = 1,yend = 1)+
    theme_bw()
}


#Calibration Chart
cal <- calibrate(f_lrm)
pdf(file=paste(output_dir, "\\calibrate_training.pdf", sep = ""),width=10,height=10)
plot(cal)
```

```
#Training set decision curve DCA
uPCX<- decision_curve(Death ~ Age+Gender+Blood_Glucose,data = be,
                                family = binomial(link ='logit'),
                                thresholds= seq(0,1, by = 0.01),
                                confidence.intervals =0.95,#95%CI
                                study.design = 'cohort')
clinicalparameters<- decision_curve(Death ~ Age+Gender+Blood_Glucose,data = be, family
= binomial(link ='logit'),
                                              thresholds= seq(0,1, by = 0.01),
                                              confidence.intervals =0.95,study.design ='cohort')
List<-list(uPCX,clinicalparameters)
plot_decision_curve(List,curve.names= c('uPCX','clinicalparameters'),
                              cost.benefit.axis =FALSE,col = c('red','blue'),
                              confidence.intervals =FALSE,standardize = FALSE)
```
